# Newborn Falls and the Need for Innovative Solutions

**DOI:** 10.1097/pq9.0000000000000881

**Published:** 2026-07-29

**Authors:** Colleen A. Hughes Driscoll, Mary Divya Kasu

**Affiliations:** From the Department of Pediatrics, University of Maryland School of Medicine, Baltimore, Md.

Falls in the pediatric setting, particularly newborn falls, have been well documented for almost 2 decades. Newborn falls, estimated to occur at a rate of 600–1,600 falls per year, pose both short- and long-term physical and psychosocial impacts on patients and their families. Despite well-characterized and consistent themes associated with newborn falls, and a 2018 safety alert from The Joint Commission, newborn falls continue to pose a challenge for families and caregivers in the hospital environment.^1^

In this month’s issue of *Pediatric Quality and Safety*, Birkinshaw et al^2^ described a quality improvement project that uses a multifaceted approach to reduce pediatric falls in a children’s hospital. “Stop the Drop” implements some commonly used interventions, including fall risk assessments, performance auditing, parent and staff education, situation-based rounding, protected rest periods, and fall debriefing, among others. Although the primary outcome, a decrease in patient falls, was sustained for just more than 2 years, compliance with specifically measured interventions was inconsistent. This finding should not be surprising when one considers the somewhat successful, but labor-intensive, approaches described in the literature. Educational reinforcements, additional rounding, and interval risk assessments have demonstrated benefit when combined with other interventions; however, they require continual determination among frontline nursing staff and hospital leaders to implement. Given the extremely low newborn fall rate^3^ and the wide array of maternal safety issues that hospital caregivers must manage on a typical obstetrical unit, continued focus on preventing newborn falls can be challenging, especially in the context of the recommended nursing staff ratios of 1:3–4 for postpartum couplet care.^4^ As “Stop the Drop” was implemented in a freestanding children’s hospital, the project was less susceptible to these challenges; thus, the observed success may not translate to units where newborn falls are most frequently observed.

The ability to maintain a high level of preventive efforts may be further affected by the recent worldwide shortage of nurses, which is now considered a global health emergency.^5,6^ In this climate, addressing newborn falls in ways that reduce or facilitate caregiver tasks is desperately needed. Over the last decade, emerging technologies have sought to prevent falls among adults. Bed exit alarms, artificial intelligence–enabled cameras that provide real-time fall risk assessments, discrete wearable sensors, remote patient monitoring, and infrared/acoustic monitoring applications have been developed to reduce falls while alerting nurses to potentially high-risk scenarios. However, the adoption of these technologies is in its early phases, and a sustained, generalizable reduction in hospital falls that does not impose alarm fatigue has yet to be demonstrated. Additionally, the primary focus of technology development has been on adults, often older people. Technology-based solutions for newborn falls will require research and development on the unique mother–baby interactions that occur in the postpartum period, most importantly, the high-risk scenario when a parent suddenly and unpredictably falls asleep while holding or feeding their newborn. Due to the low incidence of newborn falls, economic incentives to drive investment in this area may be limited without grant-based research and innovation funding. Given that childbirth is the most common reason for hospital admission, advocacy for investment in technological solutions, especially those rooted in sleep science, may be warranted.

Although parental fatigue, routinely observed during the postpartum period, is a major contributor to falls, newborn falls in nonobstetric units may occur as an unintended consequence of efforts to promote patient- and family-centered care (PFCC). Practices such as Baby Friendly and Eat, Sleep, Console aim to provide PFCC primarily through the central tenet of keeping hospitalized babies and mothers colocated as much as possible.^7–9^ Newborn falls and slips have been observed within these care models and have even extended into the Neonatal Intensive Care Unit setting, where parents may be experiencing drug-related fatigue.^8^ PFCC generally focuses on the core concepts of dignity and respect, information sharing, participation, and collaboration, with collaboration including family involvement in hospital facility design and program development.^10^ However, the extent to which family input is considered when implementing Baby Friendly and Eat, Sleep, Console programs and policies is not well described. Focused attention on family feedback during the implementation phases of these programs may identify novel strategies to reduce fall risk. Furthermore, PFCC emphasizes the importance of individualized care and, as such, may address inequities that arise from differences in support person availability during pediatric and maternal hospitalizations.

Another family-oriented aspect of newborn falls that has not been sufficiently addressed is the emotional impact on the family, particularly that of a parent directly involved in a fall or slip. Numerous quality improvement reports use an after-event review, such as a root cause analysis, as part of the improvement process. However, formal and systematic measures to address the emotional burden for parents have not been reported. Mothers may feel guilt during the postpartum hospitalization even without the occurrence of a newborn fall, especially if breastfeeding. Unfortunately, because programmatic efforts to optimize breastfeeding remain associated with newborn falls, mothers may develop additional shame following such a fall event. Investigation into strategies to reduce this emotional burden should be addressed in future studies.

The primary outcome demonstrated in “Stop the Drop” reemphasizes that fall reduction success is achievable and sustainable with commonly used care delivery implementations. Furthermore, it demonstrates the broader applicability of these interventions throughout a freestanding children’s hospital. Nevertheless, if we are to achieve comparable outcomes while easing the delivery of family-centered care, innovative strategies are required and justified.
